# Potential revival of cholinesterase inhibitors as drugs in veterinary medicine

**DOI:** 10.3389/fvets.2023.1125618

**Published:** 2023-03-02

**Authors:** Neža Žnidaršič, Malan Štrbenc, Neža Grgurevič, Tomaž Snoj

**Affiliations:** Veterinary Faculty, Institute of Preclinical Sciences, University of Ljubljana, Ljubljana, Slovenia

**Keywords:** cholinergic system, cholinesterase inhibitors, organophosphates, carbamates, canine cognitive dysfunction

## Abstract

The cholinergic system is involved in the regulation of all organ systems and has acetylcholine (ACh) as almost its only neurotransmitter. Any substance is called cholinergic if it can alter the action of acetylcholine. Cholinesterases (ChEs) are enzymes that enable the hydrolysis of acetylcholine and in this way ensure homeostasis in cholinergic synapses. Cholinesterase inhibitors (ChEi) are a group of indirect-acting cholinergic agonists that influence the activity of the cholinergic system. Several compounds that can inhibit cholinesterases are of importance to veterinary medicine from pharmacological and toxicological perspective. The frequency of their use in veterinary medicine has fluctuated over the years and is now reduced to a minimum. They are mainly used in agriculture as pesticides, and some are rarely used as parasiticides for companion animals and livestock. In recent years, interest in the use of new cholinesterase inhibitors has increased since canine cognitive dysfunction (CCD) became a recognized and extensively studied disease. Similar to Alzheimer's disease (AD) in humans, CCD can be treated with cholinesterase inhibitors that cross the blood–brain barrier. In this review, the mammalian cholinergic system and the drugs that interact with cholinesterases are introduced. Cholinesterase inhibitors that can be used for the treatment of CCD are described in detail.

## Introduction

A variety of substances are known to have inhibitory effects on cholinesterases (ChEs). Organochlorine, organophosphorus, carbamate, and other substances are known to interact with ChEs and temporarily or permanently inactivate these enzymes. Other compounds, such as divalent ions, e.g., magnesium, can also inhibit ChEs (1). These compounds were commonly used in agriculture as pesticides or insecticides in the past, so they must be recognized as a possible cause of poisoning from a clinical perspective. Unintentionally, pets, livestock, and humans can easily come into contact with them. However, their use has changed over the years. Currently, their use is limited, although the horizon is opening for some of them as medicines for canine cognitive dysfunction (CCD).

Organochlorine compounds have been used extensively for decades as very effective insecticides. They have been used in households, in agriculture, and as parasiticides in veterinary medicine, but because of their toxicity, their use for these purposes has been banned in Europe and the United States since the early 1970s, but they continue to be used in some Asian countries (2). Currently, animals or humans do not usually encounter organochlorine preparations, but because of their long half-life, organochlorines used decades ago are still present in the environment and living organisms may be exposed to low concentrations through food and drinking water. The concentration of these persistent organic pollutants is too low to affect the cholinergic system in the organism. However, prolonged exposure to low levels of organochlorine compounds has been confirmed to cause endocrine disruption (3, 4).

Organophosphorus compounds are esters of phosphoric acid. They are used in agriculture as pesticides to control insect and arthropod pests in intensive crop production. As in mammals, they inhibit acetylcholinesterase (AChE) in pests and cause their death. Although approved organophosphorus pesticides have a much shorter half-life than organochlorine compounds, animals and humans can be accidentally exposed to higher concentrations that lead to poisoning. The debate over poisoning and its treatment is outside the scope of this report and can be found elsewhere (5). On the other hand, some organophosphorus compounds are approved as parasiticides and can be used to treat pets, livestock, and bees. Organophosphorus compounds, however, include agents that act as chemical nerve agents.

Several well-described drugs (physostigmine, neostigmine, pyridostigmine, etc.) act as reversible AChE inhibitors and are carbamates. Carbamates are derived from carbamic acid. As drugs, they are used in human rather than veterinary medicine, although they can be used in non-food-producing animals. In addition, some carbamates are also used as pesticides to control insect and slug invasion and as parasiticides in veterinary medicine.

In recent years, interest in the use of new cholinesterase inhibitors (ChEi) has increased since canine cognitive dysfunction (CCD) became a recognized and extensively studied disease. Similar to Alzheimer's disease (AD) in humans, CCD can be treated with ChE inhibitors that cross the blood–brain barrier. In addition, intensive work is underway to develop agents that inhibit ChEs while having adrenergic properties, and their pharmacological properties are being investigated. It is expected that these types of agents will allow better treatment of the symptoms of AD and CCD in the future.

## Cholinergic system

The cholinergic system was named after acetylcholine (ACh), which plays the role of a neurotransmitter in the central and peripheral nervous systems. The action of ACh is expressed by binding to cholinergic (muscarinic and nicotinic) receptors in postsynaptic neurons or effector cells, while the homeostasis of cholinergic transmission is regulated by ChEs, mainly by the enzyme acetylcholinesterase (AChE) and less importantly by butyrylcholinesterase (BChE). Thus, cholinergic neurons, ACh as a neurotransmitter, cholinergic receptors, and ChEs constitute the cholinergic system (6).

### Acetylcholine (ACh)

**Figure 1:**
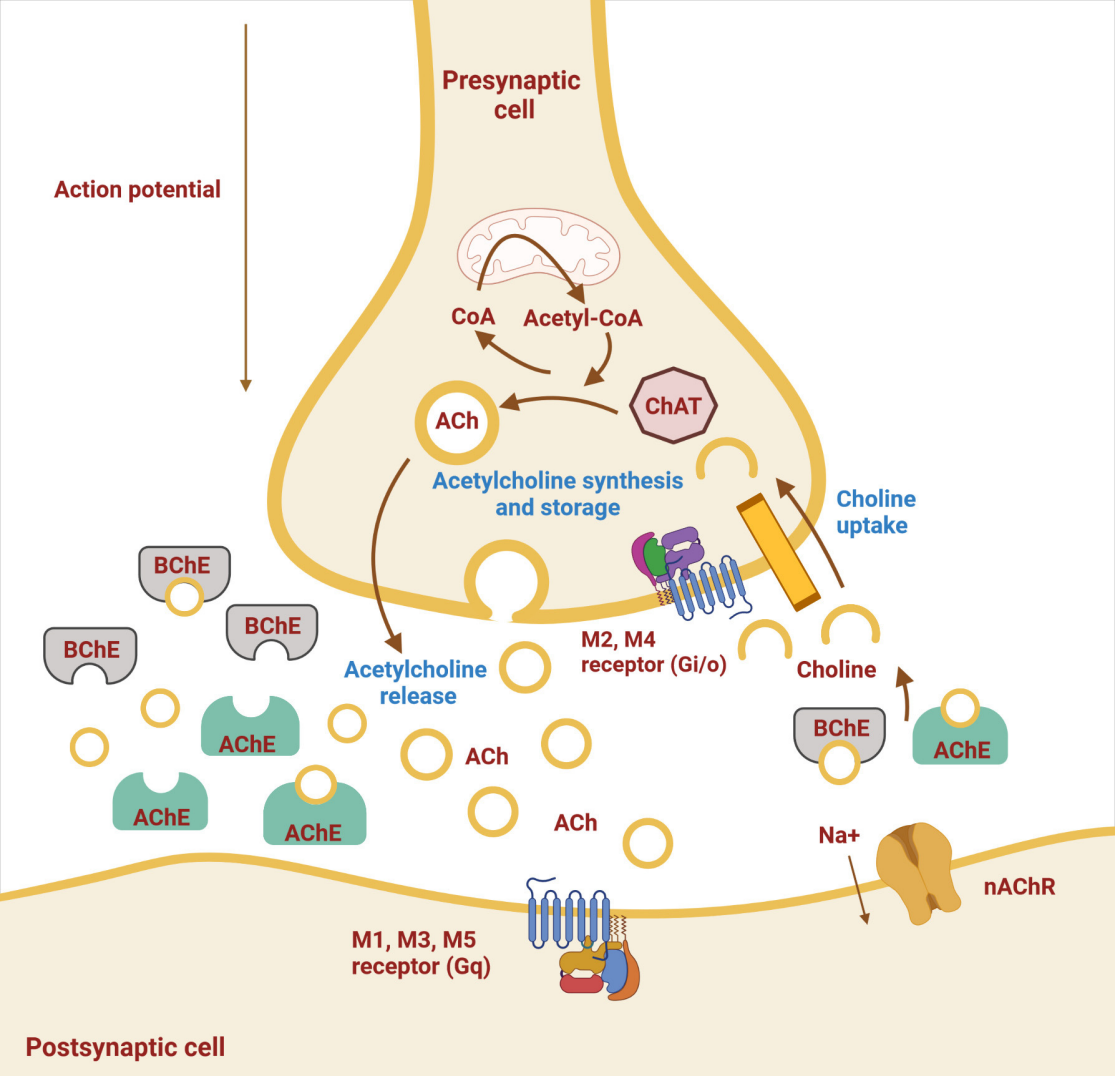
Schematic representation of a cholinergic neuron. Shown are the cell membrane of the pre- and postsynaptic neuron with synaptic cleft and ongoing processes. Synthesis of acetylcholine (ACh) by choline acetyltransferase (ChAT) from choline and acetyl-CoA, storage, and release occur in the presynaptic neuron, whereas hydrolysis of ACh by acetylcholinesterase (AChE) and butyrylcholinesterase (BChE) occurs in the synaptic cleft. The two types of cholinergic receptors in the postsynaptic membrane are muscarinic (M1–M5) and nicotinic (nAChR) receptors (Created with BioRender.com).

Although ACh had previously been synthesized in the laboratory, it was first extracted from biological material by Sir Henry Hallet Dale in 1914 (7). A few years later, Otto Loewi found that ACh was involved in the action of the vagus on heart rate and neurotransmission (8). Acetylcholine is a neurotransmitter in the cholinergic system. It provides neurotransmission in parasympathetic neurons and preganglionic sympathetic neurons at the neuromuscular junction and is widely distributed in the CNS. Thus, ACh is involved in regulating the activity of all organ systems, blood vessels, skeletal muscles, the immune system, and the brain. Acetylcholine is mainly synthesized in presynaptic neurons from choline and acetyl coenzyme A, which requires the action of choline acetyltransferase (ChAT), and is stored in intracellular vesicles. After depolarization of the presynaptic neuron, it is released into the synaptic cleft by exocytosis and provides neurotransmission (Figure 1). Acetylcholine action ends with its cleavage into choline and acetate, which occurs by AChE in the synaptic cleft. The reuptake of choline enables the synthesis of new ACh molecules in presynaptic neurons (9, 10). As shown in Table 1, ACh, as a neurotransmitter in the parasympathetic nervous system, regulates vascular tension; increases gastrointestinal motility, uterine contraction, and bronchial dilation; and decreases heart rate and blood pressure. In the sympathetic nervous system, ACh is released from preganglionic neurons and modulates synaptic transmission to postganglionic neurons, acting as a fight–flight response through noradrenaline release from axon terminals. In the CNS, ACh acts as the major excitatory neurotransmitter and regulates many cognitive functions, such as attention, learning, and memory (6).

**Table 1:** Effects on various organs of the stimulated cholinergic system.

**Effector**	**Response**	**Acetylcholine receptor**	**Reference**
CNS	Cognitive functions	mAChs	(21)
	Neuromodulation	nAChRs	
Heart	Negative chronotropic, bathmotropic, and dromotropic	M2, M4	(24)
Blood vessels	Dilatation	M1, M2, M3	(24)
	Decrease of blood pressure		
Gastrointestinal tract	Increased motility	M2, M3	(25)
	Secretion of glands		
	Sphincter relaxation		
Bronchioles	Smooth muscle contraction	M2	(26)
	Secretion of glands		
Urinary bladder	Contraction of fundus	M2, M3	(27)
	Sphincter relaxation		
Eye	Miosis, decrease of intraocular pressure	M1, M2, M3	(28)
Reproductive system	Erection		(29)
	Spermatogenesis		
	Uterine contractions		
	Gland excretion		
Sweat glands	Excretion	M3	(30)
Skeletal muscles	Contractions	nAChR	(20)
	Activity		

### Choline acetyltransferase (ChAT)

Choline acetyltransferase is the only enzyme involved in the biosynthesis of ACh; it transfers the acetyl group from acetyl-CoA to choline to form ACh. There are at least two isoforms (one common and one peripheral); alternative splicing has been studied in humans and to a lesser extent in mice and rats. Its activity is higher in motor fascicles than in sensory fascicles and is expressed in different proportions in different cortical regions and the retina. In addition to ChAT activity, the need to transport acetyl-CoA from mitochondria to the cytoplasm and intracellular choline concentrations may be rate-limiting for ACh synthesis (11).

Choline acetyltransferase activity is thought to play a central role in basic brain processes such as learning, memory, consciousness, and sleep (12). Mutations of the enzyme are the cause of congenital myasthenic syndromes (13). The amount of ChAT protein and ChAT isoforms was found to correlate with the progression of AD (14). In addition, the activities of the enzyme in plasma and/or CSF and as a marker of disease severity have been suggested but have not (yet) been tested as drug targets (15). Choline acetyltransferase is also a potential target for pharmacotherapy in several other diseases, including blood pressure disorders and cholinergic overstimulation by organophosphorus nerve agents, but a lack of useful inhibitors has limited preclinical research to date (16).

### Cholinergic receptors

The action of ACh as a neurotransmitter is mediated by binding to two types of cholinergic receptors: muscarinic (mAChR) and nicotinic (nAChR) receptors. Both families of receptors are widely distributed throughout the organism but differ in their mode of action; mAChRs are metabotropic receptors, while nAChRs are ionotropic receptors. Muscarinic receptors M1, M3, and M5 are coupled to protein Gq and activate phospholipase C, leading to the formation of diacylglycerol and inositol trisphosphate as second messengers (17). M2 and M4 are coupled to the protein Gi/o, leading to inhibition of adenylate cyclase (18). M4, as an autoreceptor, inhibits the release of ACh in the CNS (19). nAChRs are ligand-gated ionotropic receptors that regulate the transport of positively charged ions (Na^+^, K^+^, and Ca${\;}_{2}^{+}$) through membrane channels. Structurally, they are pentamers composed of five transmembrane subunits organized in a cylindrical structure with a central pore (20, 21). To date, 17 different subunits (the muscular α1-, β1-, δ-, γ-, and ε-subunits, as well as the neuronal α2–10 and β2–4 subunits) have been identified (21). nAChR subtypes occur in homomeric or heteromeric form and generally consist of both α- and β-subunits. For example, the adult muscle receptor has an α2β1ε1δ1 composition, whereas the major ganglionic subtype is α2β3, with the exception of the homomeric (α7)5 subtype, which is mainly found in the brain (22, 23). Given these different combinations of subunits, modulation of neurotransmitter release is subtype-specific, and each subtype may mediate a different physiological function; moreover, the subunits contribute to pharmacological specificity within each receptor subtype (22, 23). As described by Picciotto et al. m- and nAChRs are located pre- and postsynaptically in the CNS. Presynaptic muscarinic receptors (M2 and M4) exert inhibitory effects as autoreceptors that control further ACh release (21). Presynaptic nicotinic receptors are involved in the release of various excitatory and inhibitory neurotransmitters, such as dopamine, noradrenaline, ACh, glutamate, and GABA. Postsynaptic M2 and M4 have an inhibitory effect, whereas M1, M3, and M5 are excitatory. Postsynaptic nAChRs provide postsynaptic neuron depolarization and neurotransmission. At the neuromuscular junction, nAChRs act as ligand-gated channels to provide sodium ion transport into muscle cells, depolarization, and skeletal muscle contraction (20). In addition, ACh alters the response of neurons to the stimulation (or inhibition) of neurotransmitters and thus acts as a neuromodulator, which is particularly important for the regulation of cognitive functions (21). The effects of the cholinergic system on various organs are listed in Table 1.

### Cholinesterases

As mentioned earlier, there are two enzymes with cholinesterase activity, AChE and BChE, which hydrolyze acetylcholine into choline and acetate (31). Acetylcholinesterase and butyrylcholinesterase have a similar structure, and it is estimated that they are more than 50% identical (1, 9). However, both enzymes have their own structural features and therefore differ in their physiological roles, which will be discussed later. Each tissue contains both BChE and AChE, but in very different proportions. In the brain, BChE is expressed at a much lower level than AChE, and the change in the ratio is an important factor indicating neurodegenerative processes (31, 32).

#### Acetylcholinesterase (AChE)

Acetylcholinesterase, named after its endogenous substrate, the neurotransmitter ACh, is produced mainly in neurons but also in muscle and hematopoietic cells. Acetylcholinesterase is one of the most efficient enzymes due to its high catalytic rate. It is involved in the homeostasis of the cholinergic system in the brain, autonomic nervous system, and neuromuscular junction. Homeostasis is enabled by the hydrolysis of ACh and the termination of its action. It is also located in the membranes of red blood cells, where its role is to metabolize plasma ACh (1). In the cholinergic system, AChE is present mainly in the tetramer form, although some water-soluble dimers are also present.

**Figure 2:**
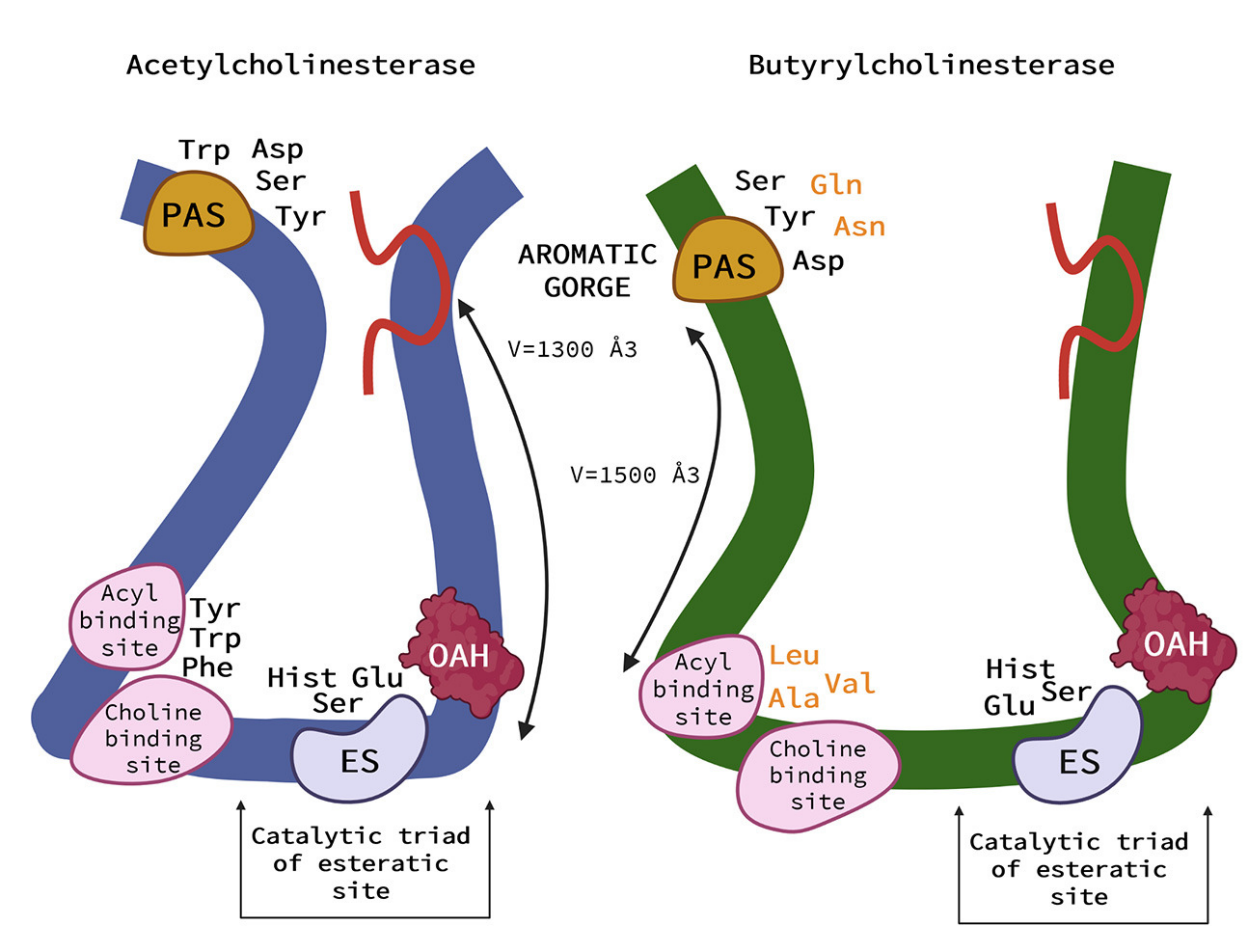
Schematic representation of the acetylcholinesterase (AChE) **(left)** and (butyrylcholinesterase) BChE **(right)**. AChE and BChE differ in the size of the binding pocket, with BChE having an approximate gorge volume of 1,500 Å3 and AChE of 1,300 Å3. Peripheral aromatic site (PAS) is located near the entrance and plays an important role in substrate entry. PAS of AChE contains serine, tyrosine, aspartic acid, and tryptophan, in BChE it is asparagine, aspartic acid, glutamine, serine, and tyrosine. The residues in BChE are more aromatic, resulting in a more negatively charged site. The acyl and choline binding sites are close to the esteratic site and consist of tryptophan, tyrosine, and phenylalanine in human AChE and are replaced by leucocin, alanine, and valine in BChE. The main role of the anionic site is to position the ligand for catalysis. Substitution with more aromatic residues leads to conformational changes that result in a deeper gorge in BChE. This allows BChE to interact with and potentially hydrolyze a much broader range of substrates and inhibitors than AChE. The catalytic triad of the esteratic site is located deep down in the gorge and consists of serine, glutamic acid, and histidine. The oxyanine hole (OAH) is a two- or three-pronged site in BChE. The Ω (omega) loop (red in this Figure) helps the enzyme “breathe”—a process that can help substrates move from the surface to deeper regions. It consists of about 30 amino acid residues (33) (Created with BioRender.com).

As is shown in Figure 2 the active site of AChE is located at the bottom of the cavity called the aromatic gorge, covered by aromatic amino acids (1, 9, 33). The active site consists of two parts. The anionic site (α anionic site) is composed of amino acids (Trp, Tyr, Phe) and interacts with the positively charged nitrogen (ammonium) in ACh, placing the ACh molecule in the correct position to bind to the nearby esteratic site of AChE (1, 9, 33). The esteratic site is another part of the active site. It contains the catalytic triad, the site where the substrate is metabolized. The catalytic triad is formed from three amino acids (Ser, His, Glu). Once ACh encounters the catalytic triad, the bond between choline and acetate is transmitted to serine, and choline is released. In addition, acetate reacts with a water molecule and releases the catalytic triad (9). On the surface of AChE, there is another important active part called the peripheral anionic site (PAS), which is also a β-anionic site. It is formed mainly by Tyr, Asp, and Trp residues. It interacts with the substrate and is located to allow the substrate to enter the gorge (34). It is believed that PAS interacts with amyloid and therefore plays an important role in the pathogenesis of AD in humans (1).

In addition, some toxins and drugs (fasciculin, berberine, donepezil, huperzine A) react with the PAS and inhibit AChE activity (1, 35, 36). However, because the catalytic rate of AChE is one of the most active in the organism, it could be inhibited by an excess of substrate (1). Despite the primary function of AChE, researchers have found that AChE also plays a role in neuronal development. Embryologically, it is involved in nervous system development and is expressed by developing neurons and during periods of axonal growth (31).

#### Butyrylcholinesterase (BChE)

Butyrylcholinesterase (BChE), formerly called “pseudocholinesterase,” is produced mainly by the liver (33). The richest sources of BChE enzymes in the body are plasma and liver, as well as leg muscles and skin (37). Although BChE can metabolize ACh, it has a higher affinity for butyrylcholine (BCh), a synthetic compound that does not occur naturally in the body and is a choline-based ester with neurotransmitter activity. It is used as a clinical laboratory tool to distinguish between enzymes (33). The main structural difference between BChE and AChE is that 6 of the 14 aromatic amino acids lining the active site gorge of AChE are replaced by aliphatic residues in BChE (33). These conformational changes result in a larger gorge volume in BChE, which allows BChE to interact with and potentially hydrolyze a much broader range of substrates than AChE. Butyrylcholinesterase is therefore referred to as the promiscuous or non-specific “bigger brother” of the smaller and much more specific AChE (33, 38).

Although ChEs share some of these functions, the biological role of BChE has a much more obscure purpose. The higher concentration of BChE in plasma led to the theory that BChE evolved from AChE to be a general detoxifying agent while still retaining some function in the process of neurotransmission (33, 37). However, it is known that it has no function that could not be compensated for by other enzymes (37). On the other hand, BChE is known to play an important role in drug detoxification (examples: physostigmine, cocaine, aspirin, succinylcholine, mivacurium, heroin) (31, 33, 39). Moreover, BChE restores cholinergic transmission when AChE activity is impaired or absent, e.g., in the brains of AD patients (9). This was suggested in studies using the drugs donepezil and huperzine A in BChE knockout mice (39). Similar changes in the physiological functions of both enzymes have been observed in humans, where AChE concentration decreases and BChE concentration increases with the progression of AD, increasing the ratio of BChE to AChE from 0.2 to up to 11. In addition, BChE may be involved in lipid metabolism and the development of inflammation (37).

### Drugs that inhibit ChE activity

Several compounds can bind to ChEs and inhibit their activity, and only a few compounds are used as drugs affecting the cholinergic system of mammals. To achieve the desired pharmacological effect, these drugs must inhibit ChEs for a short period of time so that they are spontaneously hydrolyzed after binding to active sites in ChEs. In addition to drugs that interact with animal ChEs, drugs that interact with the cholinergic system and are used as parasiticides are relatively common in veterinary medicine. Since there is a possibility of interaction with the host AChE, these drugs are also important from a toxicological point of view.

### Carbamates

The effects of these classic drugs are well-described in the literature. Carbamates are reversible inhibitors of ChEs and block the catalytic site of AChE, resulting in decreased activity of AChE and thus increased concentration of ACh in the synapses and neuromuscular junction and prolonged cholinergic action. Therefore, they are indirect-acting cholinergic agonists. The effects of carbamates can be predicted in terms of the anatomic location of muscarinic and nicotinic receptors and pharmacokinetic properties. However, treatment with neostigmine and physostigmine may lead to adverse effects because these compounds stimulate preganglionic sympathetic nerves and adrenal glands and may produce unexpected effects by releasing catecholamines (40).

#### Edrophonium

Edrophonium is a small molecule containing a positively charged nitrogen ion that forms an electrostatic bond with the anionic site of ChEs. Since the anionic site is blocked by edrophonium, the natural substrate cannot reach the esteratic (catalytic) site of the enzyme. This inhibits the activity of the ChEs for a short time until the electrostatic force between the ions is removed when the ChEs are reactivated (40). Unlike other carbamates, edrophonium is not hydrolyzed, but the anionic site is spontaneously released from the drug. Edrophonium is used for diagnostic procedures in myasthenia gravis-like disorders primarily in dogs and cats (41). Short-term administration of edrophonium improves skeletal muscle weakness in myasthenia or myasthenia-like disorders (42).

#### Physostigmine

Physostigmine is a tertiary amine that reversibly blocks ChEs. Transient binding between ChEs and physostigmine occurs at the esteratic site of the enzyme. It is then hydrolyzed, and the ChEs are reactivated. It has few indications and is rarely used in veterinary medicine. It has been reported that physostigmine can be used to treat glaucoma in dogs and cats (43), but safer drugs such as carbonate anhydrase inhibitors or some beta-blockers (timolol) are more commonly used for this purpose. Physostigmine crosses the blood–brain barrier and can be used to treat cognitive dysfunction and intoxication with atropine, scopolamine, or belladonna. It has been reported that physostigmine can be used for symptomatic treatment of ivermectin toxicosis in collies (44). Theoretically, rumen or intestinal atony can be treated with physostigmine in ruminants, but the drug is not approved for use in food-producing animals. Physostigmine can also be used to prevent organophosphate (or chemical weapon) poisoning. Administration of sublethal doses of physostigmine (via the skin or eyes) protects against lethal doses of organophosphorus compounds such as soman and sarin, which are used as chemical weapons (45, 46).

#### Neostigmine

Administration of neostigmine acts more intensely on the neuromuscular junction than on the cholinergic transmission that activates muscarinic receptors in the digestive system. Because it is a quaternary nitrogen compound, it does not cross the blood–brain barrier and does not exhibit central effects. However, it has been described to treat rumen atony or caecal dislocation without torsion in ruminants (47). It can also be used as an antidote in curare-like poisoning (48). Neostigmine preparations are not approved for veterinary use, which limits their use in livestock. After general anesthesia, it is used in dogs to rapidly interrupt neuromuscular blockade caused by non-depolarizing muscle relaxants (49).

#### Pyridostigmine

Pyridostigmine is a carbamate containing quaternary ammonium. It binds to the catalytic site of ChEs and temporarily blocks their activity. Its action is in the peripheral nervous system, as the positively charged ammonium ion can cross the blood–brain barrier (50). Pyridostigmine is a drug of choice for the prevention of the nerve agent intoxication and treatment of myasthenia gravis-like disease in dogs and cats (51, 52). Because it is not approved for veterinary use, human preparations, usually in syrup and tablet form, are used. The recommended dose is given in Table 2, but the dose can be individualized depending on the stage of the disease.

**Table 2:** Use of drugs that act as AChE inhibitors in animals.

**Drug**	**Animal species**	**Dose (mg/kg) Route of administration**	**Indication**	**Reference**
Edrophonium chloride	Dogs and cats	0.1–0.2 IV	Diagnosis of myasthenia-like diseases	(42)
Physostigmine	All species	Individual regimen	Atropine intoxication	(50)
Physostigmine	Dogs	2.5 mcg/kg/h	Prevention of nerve agent intoxication	(45, 46, 51)
Physostigmine	Dogs and cats	Eye drops 2%	Glaucoma	(43)
Physostigmine	Cattle	0.04–0.08 IV	Curare-like intoxications (e.g., *Delphinium barbeyi*)	(48)
Pyridostigmine	Dogs and cats	0.5 Orally	Myasthenia gravis	(52)
Neostigmine	Cattle	0.02 SC	Rumen atony	(47)
Neostigmine	Sheep	0.01–0.02 SC	Rumen atony	(47)
			Caecal dislocation	
Neostigmine	Cattle	87.5 mg in 10 L of sodium-glucose infusion: 2 drops/s	Caecal dislocation	(47)
Neostigmine	Cattle	To the effect	Curare and curare-like intoxications	(48)
Neostigmine	Dogs	0.02–0.07 IV	To terminate myorelaxation during anesthesia	(49)

### Organophosphates and carbamates as parasiticides

Although the main era of using organophosphates and carbamates as parasiticides has already passed, some of them are still approved in some countries for use in veterinary medicine. Approved organophosphates are used as parasiticides for the control of ecto- and endoparasites. Trichlorfon is a pro drug that is metabolized to dichlorvos after administration. Both of them were used to control nematodes and ectoparasites, mainly in dogs (53). Additionally, coumaphos is used for the treatment of bee colonies against infestation with Varroa destructor. Since it accumulates in beeswax after each use, it should be used sparingly (54). Additionally, two carbamate drugs, carbaril and propoxur, are used for the control of ectoparasites in pets and livestock. It is expected that these organophosphate and carbamate drugs interact with the cholinergic system of the parasites and do not affect the host; thus, their characteristics are not a topic of this review. However, it must be mentioned that in addition to organophosphates and carbamates as veterinary drugs, they are also used as pesticides in agriculture, and accidental contact of animals with these compounds can cause severe intoxication due to AChE blockage (5, 55).

### Existing acetylcholinesterase inhibitors for the treatment of cognitive impairment

Rivastigmine, galantamine, and donepezil are AChE inhibitors approved for the treatment of Alzheimer's disease (AD). Their use is considered suboptimal therapy because they do not halt disease progression but only improve symptoms that lead to better cognitive function (56). Based on research and literature, there are limited data on the clinical use of these drugs in animals, particularly in dogs with cognitive impairment. Matsunami et al. reported that “canine cognitive dysfunction syndrome criteria” improved after a 2-week study with donepezil hydrochloride (57). In the study by Araujo et al. the role of cholinergic hypofunction in the canine model was investigated (58). In two tasks, a delayed non-matching-to-position (DNMP) task to assess working memory and an oddity-discrimination task to assess complex learning, they examined the effect of the cholinesterase inhibitors phenserine and donepezil on performance in older dogs. Phenserine (0.5 mg/kg; orally) and donepezil (1.5 mg/kg; orally) significantly improved performance (58). Phenserine is an AChE inhibitor whose research was abandoned after clinical phase III due to some methodological problems (59). Out of curiosity, we searched the commercial website for the clinical use of AChE inhibitors in dogs. One website recommends and sells donepezil 23 and 10 mg tablets under the brand name Aricept for dogs with CCD. This drug is otherwise approved for human use in the United States (60). We could not find any clear information or reports from the FDA or EMA on its use in dogs.

## Use of ChE inhibitors for the treatment of canine cognitive dysfunction

Acetylcholine is also responsible for learning and memory formation (9, 61). The pathological processes in Alzheimer disease (AD) lead to a deficiency of ACh and consequent loss of cholinergic neurons. Neurotransmission is therefore impaired, leading to cognitive deficits (56). This so-called cholinergic hypothesis is the basis for treating AD with drugs that increase ACh levels. Cholinergic hypofunction is also present in dogs with CCD (62, 63). Cognitive dysfunction in dogs is defined as a chronic progressive neurodegenerative disease that affects 14–60% of old dogs but is diagnosed in less than 2% of all affected individuals (64). It most commonly occurs in dogs older than 8 years and results in changes in behavior and cognitive abilities (65). Dogs also show age-related cognitive impairments that parallel the behavioral symptoms of AD. In addition to behavioral symptoms, the neuropathology of both diseases is similar in several aspects (63). For this reason, dogs with CCD represent one of the potentially most important animal models for studying the treatment of AD due to their functional and neuroanatomical similarity to humans (66, 67).

As mentioned earlier, phenserine and donepezil have been successfully tested in dogs (58). In another study by Studzinski et al. the efficacy of two AChE inhibitors—CP-118,954 and phenserine—was tested in a modified delayed non-matching to position task (68). Minimal cognitive enhancing effects were seen after treatment with CP-118,954. In contrast, phenserine was able to improve learning and memory in dogs and showed positive effects on memory. In humans, CP-118,954 was discontinued after reaching clinical phase II, as was the case with phenserine (59, 68).

More important, however, is focusing on the area that most of the ChE research has focused on in the last two decades: the inhibition of one or both enzymes, AChE and BChE, by different compounds from different chemical groups. The reason is the almost desperate search for a drug that could improve the symptoms of AD and CCD. Recently, a new series of selective nanomolar BChE inhibitors have been developed (61, 69). The rationale for the development of selective BChE inhibitors is that during the progression of AD and CCD, cholinergic transmission, and AChE activity decrease. To compensate, BChE takes over and terminates up to 90% of cholinergic signaling in the brain. In addition, these agents have no peripheral parasympathomimetic side effects, which are quite common with AChE inhibitors due to the selective inhibition of BChE (61). In the last clinical trial, dogs with CCD were treated with newly developed BChE inhibitors (69, 70). Their cognitive abilities were assessed using a questionnaire (CADES score) and two cognitive performance tests: Food search test and Problem Solving Test. The dogs participated in therapy for at least 6 months, and the dogs with moderate cognitive impairment showed improvement in clinical signs and performance-related tests compared to the untreated group (*p* < 0.001). In addition, owners reported improved quality of life for the treated dogs and for themselves. This argues for the use of cholinergic medications in older dogs and allows for future screening of therapeutics for AD (70).

The lack of information on clinical trials of CCD with ChEi is the main reason why we cannot make general conclusions or suggestions about the efficacy and safety of these drugs. Several studies on this problem are expected to be conducted in the next few years, and the issue of CCD treatment will become clearer.

## Drug interactions

It is well-known that some drugs may potentiate or inhibit the action of cholinesterase inhibitors. Thus, atropine counteracts the muscarinic actions of physostigmine, neostigmine, and pyridostigmine; therefore, atropine decreases the action of carbamates (pyridostigmine), and conversely, carbamates block the action of atropine (50). The interaction of carbamates and depolarizing myorelaxants (succinylcholine) results in enhanced and prolonged myorelaxation. The effect of non-depolarizing myorelaxants is shortened or reversed by administration of neostigmine and other carbamates. This combination therapy is used for faster recovery after the use of myorelaxants such as pancuronium or rocuronium for myorelaxation during anesthesia (71). In addition, some antibiotics, e.g., aminoglycosides, polymyxins, tetracyclines, and bacitracin, can prolong neuromuscular blockade when used with non-depolarizing myorelaxants (50, 72). Adrenergic beta inhibitors may attenuate the effect of pyridostigmine in the treatment of myasthenia gravis. As mentioned earlier, pretreatment with carbamates can prevent poisoning and severe effects of chemical weapons such as sarin, soman, etc. (45, 46, 51).

## Safety and adverse effects of cholinesterase inhibitors (ChEi)

One of the main disadvantages of approved ChEi is its frequent side effects. This is due to an increased total amount of ACh caused by inhibition of ChEs not only in the brain but also in the peripheral nervous system, parasympathetic autonomic nervous system, and basal ganglia. In humans, gastrointestinal side effects, such as diarrhea, vomiting, and nausea, are the most common signs of an overstimulated parasympathetic nervous system (69, 73). In addition to gastrointestinal issues, other common side effects cited include dizziness, headache, and weight loss (73). With regard to donepezil, sleep disturbances have been reported with its use, and there are case reports of cardiac side effects. However, studies to date have not shown serious arrhythmogenic, hypotensive, or negative chronotropic effects (73). Such symptoms may also occur in patients with mild AD but may increase in severity and frequency as the disease progresses. Their frequency and intensity may depend on the dose and route of administration (73). Nevertheless, the three marketed cholinesterase inhibitors are considered safe and remain the first choice in AD therapy (74). However, little is known about the safety and proper dosing of these drugs in animals, particularly dogs (62). In Araujo's study, side effects such as tremors, vomiting, and hypersalivation occurred in dogs receiving 6 mg/kg donepezil. Side effects occurred less frequently in the placebo and 1.5 mg/kg groups (58). Dogs treated with medium and high doses of CP-118,954 showed slightly impaired performance in behavioral tests, probably due to side effects (68). At the same time, no side effects have been reported with the use of phenserine, although it is possible that they may occur with higher doses and prolonged administration (58, 68).

In a clinical trial with a newly developed BChE inhibitor, Košak et al. and Zakošek Pipan et al. reported that the timing of treatment may be critical (69, 70). In their study, vomiting and nausea occurred in all dogs with severe cognitive impairment after the beginning of treatment. In any case, no side effects were observed when using the new BChEi to treat dogs exhibiting moderate cognitive impairment. Therefore, the new inhibitor is unlikely to have muscarinic effects in the early stages of the disease. The same observation was made in a preclinical study in mice, in which the newly developed BChEi had no negative effects on the motor coordination of the mice and did not cause motor deficits. Moreover, no acute cholinergic side effects were observed even at a high dose (100 mg/kg) in mice (69, 70).

Finally, the differential effects of dose and timing indicate the need for comprehensive pharmacokinetic and pharmacodynamic profiling in target animals before veterinary use can be considered (58).

## Discussion

The time when some cholinesterase inhibitors such as organochlorines were used as veterinary drugs is certainly over. Since other effective drugs are currently available, organophosphorus and carbamate parasiticides are not used as often as they were. However, organophosphates and carbamates are still used in agriculture as pesticides, so there is a possibility of accidental poisoning if animals come into contact with them. Carbamate drugs are used primarily for diagnostic purposes and are more widely used in human medicine. Nevertheless, edrophonium can be used for diagnostic purposes in myasthenia gravis-like disorders, especially in dogs and cats (41), and physostigmine can be used to treat glaucoma in dogs and cats (43) and for antimuscarinic toxicity. Pyridostigmine and neostigmine are the most used AChE inhibitors for the treatment of myasthenia gravis, but pyridostigmine is preferred because of its longer duration of action and lower potential for adverse gastrointestinal effects (52). However, some ChE inhibitors are gaining importance because their mechanism of action plays a role in neurodegenerative diseases.

Alzheimer' disease is one of the most common causes of dementia in the elderly and a major public health concern in the world. In veterinary medicine, CCD is also a problem in older dogs and is becoming more well-known. Researchers are trying to develop new therapeutics to alleviate the symptoms of this neurodegenerative disorder. In addition, old dogs (and, more recently, cats) have been recognized as animal models for AD (75). The main advantage over non-transgenic aged rodent species is that dogs are more similar to humans from a neuroanatomical and physiological perspective (63, 67), which means that they spontaneously develop neurodegenerative diseases, unlike rodents. In addition, the use of multiple animal models could also be crucial for safety reasons. This was evident in the AN-1792 clinical trials that were initiated in humans based on positive findings from the transgenic mouse model. The trials were stopped after 18 patients developed meningoencephalitis, some of whom died. The preclinical studies had not predicted this adverse event, probably because the Aβ vaccine in the transgenic mouse targeted a foreign peptide, whereas in humans, it targeted an endogenous peptide and triggered an autoimmune response. Perhaps this result could have been avoided if the preclinical data had also been obtained from primates or dogs that naturally deposit amyloids (68, 76). As a result, there is increasing interest in the use of new ChE inhibitors. It has been reported that approved AChE inhibitors for AD have been tested in dogs (58, 68), but these studies are not supported by new trials, and little is reported on their clinical use. In most cases, other drugs are used for symptomatic treatment. However, some BChE inhibitors have been tested recently (61, 69, 70, 77). It is recommended to focus only on BChE inhibition as a target for symptomatic treatment because it offsets the diminishing effect of AChE in nervous tissue and fewer side effects have been reported compared with AChE inhibitors. Owners have reported great satisfaction with the tested drug, as it has improved their dogs' lives and their own (70). In view of the above findings, the new BChEi shown in our study may be a good alternative to the currently available AD and CCD treatments (70).

## Future prospects

The way to approve drugs for the treatment of CCD is still long, but there is hope as the research and development of new chemical mediators progresses. Recently, a new series of BChE inhibitors have been optimized (77). Additionally, the development of new compounds, the so-called multitarget direct ligands, is very interesting and encouraging. They target multiple signaling pathways in the neurodegenerative process present in AD and CCD and could not only alleviate symptoms but also counteract cellular processes associated with neurodegeneration (74, 78).

The treatment of canine cognitive dysfunction with ChEi in veterinary medicine is, in fact, the only new application we have been able to glean from recent publications. Considering the increasing awareness of a better quality of life of geriatric pets, we can assume that the number of drugs considered for this purpose will increase and that other age-related treatable pathologies will probably be recognized, which prompted us to write the current review. Although a very similar neurodegenerative syndrome has also been recognized in feline patients, no (successful) attempt has been made to treat them with ChE inhibitors (75, 79, 80).

Finally, we must not forget that CCD is still an underdiagnosed disease. The reason for this is the inability of owners to recognize the disease in its early stages, the inexperience of veterinarians, and the lack of diagnostic tools, which too often leads to symptoms being mistaken for the normal aging process. However, early detection of cognitive aging disorders in older pets is of paramount importance. The fact that dogs are good models for AD opens up the possibility of testing new drugs in dogs and treating their cognitive issues. Another thing to consider is that pets are exposed to similar environmental risk factors as humans, and one could even say that we lead the same lifestyle. This could bring many interesting questions and answers in the future.

## Author contributions

NŽ: data selection, preparation of the figures, writing, and editing. MŠ and NG: review and editing. TS: design of the manuscript, data selection, writing, review, and editing. All authors contributed to the article and approved the submitted version.

## References

[1] Pohanka M. Cholinesterases, a target of pharmacology and toxicology. Biomed Pap Med Fac Univ Palacky Olomouc Czech Repub. (2011) 155:219–29. 10.5507/bp.2011.036 22286807

[2] Jayaraj R Megha P Sreedev P. Organochlorine pesticides, their toxic effects on living organisms and their fate in the environment. Interdiscip Toxicol. (2016) 9:90–100. 10.1515/intox-2016-0012 28652852 PMC5464684

[3] Kelce WR Stone CR Laws SC Gray LE Kemppainen JA Wilson EM. Persistent DDT metabolite p,p'-DDE is a potent androgen receptor antagonist. Nature. (1995) 375:581–85. 10.1038/375581a0 7791873

[4] Martyniuk CJ Mehinto AC Denslow ND. Organochlorine pesticides: agrochemicals with potent endocrine-disrupting properties in fish. Mol Cell Endocrinol. (2020) 507:110764. 10.1016/j.mce.2020.110764 32112812 PMC10603819

[5] Gupta RC Milatovic D. Organophosphates and carbamates. In:Gupta RC, editor. Veterinary Toxicology. 2nd ed. London: Elsevier (2012) p. 573–85.

[6] Bertrand D Wallace TL. A review of the cholinergic system and therapeutic approaches to treat brain disorders. Curr Top Behav Neurosci. (2020) 45:1–28. 10.1007/7854_2020_141 32451956

[7] Tansey EM. Henry Dale and the discovery of acetylcholine. C R Biol. (2006) 329:419–25. 10.1016/j.crvi.2006.03.012 16731499

[8] McCoy AN Tan SY. Otto Loewi (1873–1961): dreamer and nobel laureate. Singapore Med J. (2014) 55:3–4. 10.11622/smedj.2014002 24452970 PMC4291908

[9] Moodie LWK Sepčić K Turk T FrangeŽ R Svenson J. Natural cholinesterase inhibitors from marine organisms. Nat Prod Rep. (2019) 36:1053–92. 10.1039/c9np00010k 30924818

[10] Taylor P Brown JH. Synthesis, storage and release of acetylcholine. In:Siegel GJ Agranoff BW Albers RW Fisher SK Uhler MD, editors. Basic Neurochemistry: Molecular, Cellular and Medical Aspects. Philadelphia, PA: Lippincott-Raven (1999). p. 192–94.

[11] Deutch AY Roth RH. Pharmacology and biochemistry of synaptic transmission. In: Byrne J, Heidelberger, Waxham M. From Molecules to Networks. An Introduction to Cellular and Molecular Neuroscience. Cambridge, MA: Academic Press (2014) p. 207–37.

[12] Witzemann V. Choline acetyltransferase. In:Enna SJ Bylund BD, editors. xPharm: The Comprehensive Pharmacology Reference. Amsterdam; Boston, MA: Elsevier (2007) p. 1–5. 10.1016/b978-008055232-3.6052

[13] Ohno K Tsujino A Brengman JM Harper CM Bajzer Z Udd B. Choline acetyltransferase mutations cause myasthenic syndrome associated with episodic apnea in humans. Proc Natl Acad Sci USA. (2001) 98:2017–22. 10.1073/pnas.98.4.2017 11172068 PMC29374

[14] Gill SK Ishak M Dobransky T Haroutunian V Davis KL Rylett RJ. 82-kDa choline acetyltransferase is in nuclei of cholinergic neurons in human CNS and altered in aging and Alzheimer disease. Neurobiol Aging. (2007) 28:1028–40. 10.1016/j.neurobiolaging.2006.05.011 16797789

[15] Karami A Darreh-Shori T Schultzberg M Eriksdotter M. CSF and plasma cholinergic markers in patients with cognitive impairment. Front Aging Neurosci. (2021) 13:704583. 10.3389/fnagi.2021.704583 34512307 PMC8426513

[16] Wiktelius D Allgardsson A Bergström T Hoster N Akfur C Forsgren N. In situ assembly of choline acetyltransferase ligands by a hydrothiolation reaction reveals key determinants for inhibitor design. Angew Chem Int Ed Engl. (2021) 60:813–19. 10.1002/anie.202011989 33079431

[17] Caulfield MP Birdsall NJ. International Union of Pharmacology. XVII. Classification of muscarinic acetylcholine receptors. Pharmacol Rev. (1998) 50:279–90. 9647869

[18] Starke K Göthert M Kilbinger H. Modulation of neurotransmitter release by presynaptic autoreceptors. Physiol Rev. (1989) 69:864–989. 10.1152/physrev.1989.69.3.864 2568648

[19] Tzavara ET Bymaster FP Davis RJ Wade MR Perry KW Wess J. M4 muscarinic receptors regulate the dynamics of cholinergic and dopaminergic neurotransmission: relevance to the pathophysiology and treatment of related CNS pathologies. FASEB J. (2004) 18:1410–2. 10.1096/fj.04-1575fje 15231726

[20] Martyn JA Fagerlund MJ Eriksson LI. Basic principles of neuromuscular transmission. Anaesthesia. (2009) 1:1–9. 10.1111/j.1365-2044.2008.05865.x 19222426

[21] Picciotto MR Higley MJ Mineur YS. Acetylcholine as a neuromodulator: cholinergic signaling shapes nervous system function and behavior. Neuron. (2012) 76:116–29. 10.1016/j.neuron.2012.08.036 23040810 PMC3466476

[22] Rang HP Ritter JM Flower RJ Henderson G Dale MM. Cholinergic transmission. In:Ritter J Flower R Henderson G Loke YK MacEwan D Rang H, editors. Rang and Dale's Pharmacology. Edinburgh: Elsevier, Churchill Livingstone (2020) p. 175–96.

[23] Rang HP Ritter JM Flower RJ Henderson G Dale MM. Other transmitters and modulators. In:Ritter J Flower R Henderson G Loke YK MacEwan D Rang H, editors. Rang and Dale's Pharmacology. Edinburgh: Elsevier, Churchill Livingstone (2020) p. 499–513.

[24] Saternos HC Almarghalani DA Gibson HM Meqdad MA Antypas RB Lingireddy A. Distribution and function of the muscarinic receptor subtypes in the cardiovascular system. Physiol Genomics. (2018) 50:1–9. 10.1152/physiolgenomics.00062.2017 29093194

[25] Tanahashi Y Komori S Matsuyama H Kitazawa T Unno T. Functions of muscarinic receptor subtypes in gastrointestinal smooth muscle: a review of studies with receptor-knockout mice. Int J Mol Sci. (2021) 22:926. 10.3390/ijms22020926 33477687 PMC7831928

[26] Coulson FR Fryer AD. Muscarinic acetylcholine receptors and airway diseases. Pharmacol Ther. (2003) 98:59–69. 10.1016/s0163-7258(03)00004-4 12667888

[27] Chess-Williams R. Muscarinic receptors of the urinary bladder: detrusor, urothelial and prejunctional. Auton Autacoid Pharmacol. (2002) 22:133–45. 10.1046/j.1474-8673.2002.00258.x 12452898

[28] Nietgen GW Schmidt J Hesse L Hönemann CW Durieux ME. Muscarinic receptor functioning and distribution in the eye: molecular basis and implications for clinical diagnosis and therapy. Eye (Lond). (1999) 13:285–300. 10.1038/eye.1999.78 10624421

[29] Avellar MC Lázari MF Porto CS. Expression and function of G-protein-coupled receptors in the male reproductive tract. An Acad Bras Cienc. (2009) 81:321–44. 10.1590/s0001-37652009000300002 19722007

[30] Schiavone A Brambilla A. Muscarinic M3 receptors mediate secretion from sweat glands in the rat. Pharmacol Res. (1991) 23:233–9. 10.1016/s1043-6618(05)80082-9 2068048

[31] Lockridge O Quinn DM. Esterases. In:McQuenn CA, editor. Comprehensive Toxicology. Kidlington: Elsevier (2010) p. 245–50.

[32] Mushtaq G Greig NH Khan JA Kamal MA. Status of acetylcholinesterase and butyrylcholinesterase in Alzheimer's disease and type 2 diabetes mellitus. CNS Neurol Disord Drug Targets. (2014) 13:1432–9. 10.2174/1871527313666141023141545 25345511 PMC5878042

[33] De Boer D Nguyen N Mao J Moore J Sorin EJ. A comprehensive review of cholinesterase modeling and simulation. Biomolecules. (2021) 11:580. 10.3390/biom11040580 33920972 PMC8071298

[34] Branduardi D Gervasio FL Cavalli A Recanatini M Parrinello M. The role of the peripheral anionic site and cation-pi interactions in the ligand penetration of the human AChE gorge. J Am Chem Soc. (2005) 127:9147–55. 10.1021/ja0512780 15969593

[35] Colović MB Krstić DZ Lazarević-Pašti TD BondŽić AM Vasić VM. Acetylcholinesterase inhibitors: pharmacology and toxicology. Curr Neuropharmacol. (2013) 11:315–35. 10.2174/1570159X11311030006 24179466 PMC3648782

[36] Eastman J Wilson EJ Cerveñansky C Rosenberry TL. Fasciculin 2 binds to the peripheral site on acetylcholinesterase and inhibits substrate hydrolysis by slowing a step involving proton transfer during enzyme acylation. J Biol Chem. (1995) 270:19694–701. 10.1074/jbc.270.34.19694 7649979

[37] Lockridge O. Review of human butyrylcholinesterase structure, function, genetic variants, history of use in the clinic, and potential therapeutic uses. Pharmacol Ther. (2015) 148:34–46. 10.1016/j.pharmthera.2014.11.011 25448037

[38] Saxena A Redman AM Jiang X Lockridge O Doctor BP. Differences in active site gorge dimensions of cholinesterases revealed by binding of inhibitors to human butyrylcholinesterase. Biochemistry. (1997) 36:14642–51. 10.1021/bi971425+ 9398183

[39] Duysen EG Li B Darvesh S Lockridge O. Sensitivity of butyrylcholinesterase knockout mice to (–)-huperzine A and donepezil suggests humans with butyrylcholinesterase deficiency may not tolerate these Alzheimer's disease drugs and indicates butyrylcholinesterase function in neurotransmission. Toxicology. (2007) 233:60–9. 10.1016/j.tox.2006.11.069 17194517

[40] Adams RH. Introduction to neurohumoral transmission and the autonomic nervous system. In:Riviere JE Papich MG, editors. Veterinary Pharmacology and Therapeutics. Ames, IA: Wiley-Blackwell (2009). p. 101–23.

[41] Mignan T Targett M Lowrie M. Classification of myasthenia gravis and congenital myasthenic syndromes in dogs and cats. J Vet Intern Med. (2020) 34:1707–17. 10.1111/jvim.15855 32668077 PMC7517852

[42] Khorzad R Whelan M Sisson A Shelton GD. Myasthenia gravis in dogs with an emphasis on treatment and critical care management. J Vet Emerg Crit Care. (2011) 21:193–208. 10.1111/j.1476-4431.2011.00636.x 21631705

[43] Maślanka T. Autonomic drugs in the treatment of canine and feline glaucoma–Part I: medications that lower intraocular pressure by increasing the outflow of aqueous humour. Pol J Vet Sci. (2014) 17:741–52. 10.2478/pjvs-2014-0110 25638993

[44] Tranquilli WJ Paul AJ Seward RL Todd KS Dipietro JA. Response to physostigmine administration in collie dogs exhibiting ivermectin toxicosis. J Vet Pharmacol Ther. (1987) 10:96–100. 10.1111/j.1365-2885.1987.tb00083.x 3586129

[45] Kim WS Cho Y Kim JC Huang ZZ Park SH Chois EK. Protection by a transdermal patch containing physostigmine and procyclidine of soman poisoning in dogs. Eur J Pharmacol. (2005) 525:135–42. 10.1016/j.ejphar.2005.09.052 16256978

[46] Bonhage MR Chilcoat CD Li Q Melendez V Flournoy WS. Evaluation of two scopolamine and physostigmine pretreatment regimens against nerve agent poisoning in the dog. J Vet Pharmacol Ther. (2009) 32:146–53. 10.1111/j.1365-2885.2008.01013.x 19290944

[47] Coetzee JHF. Drugs for specific purposes in the ruminant digestive system. In: MSD Manual, Veterinary Manual. (2016). Available online at: https://www.msdvetmanual.com/pharmacology/systemic-pharmacotherapeutics-of-the-digestive-system/drugs-for-specific-purposes-in-the-ruminant-digestive-system (accessed October 26, 2022).

[48] Pfister JA Panter KE Manners GD Cheney CD. Reversal of tall larkspur (*Delphinium barbeyi*) poisoning in cattle with physostigmine. Vet Hum Toxicol. (1994) 36:511–4. 7900266

[49] Martin-Flores M Lorenzutti AM Litterio NJ Rossetti VL Zarazaga MP Bonetto CC. Speed of reversal of vecuronium neuromuscular block with different doses of neostigmine in anesthetized dogs. Vet Anaesth Analg. (2017) 44:28–34. 10.1111/vaa.12395 27258375

[50] Adams RH. Cholinergic pharmacology: autonomic drugs. In:Riviere JE Papich MG, editors. Veterinary Pharmacology and Therapeutics. Ames, IA: Wiley-Blackwell (2009). p. 157–79.

[51] Meshulam Y Cohen G Chapman S Alkalai D Levy A. Prophylaxis against organophosphate poisoning by sustained release of scopolamine and physostigmine. J Appl Tox. (2001) 21:75–8. 10.1002/jat.815 11920924

[52] McMurphy MR Davis GE Ranking JA Marjory AA Lutjemeier JB Kenney JM. Cholinergic pharmacology: autonomic drugs. In: Riviere EJ, Papich GM. Veterinary Pharmacology and Therapeutics. Hoboken, NJ: John Wiley and Sons, Inc. (2018). p. 151–64.

[53] Sarchahi AA. Effect of Trichlorfon (Neguvon) against *Sarcoptes scabiei var canis*. J Appl Anim Res. (2005) 28:15–6. 10.1080/09712119.2005.9706780

[54] Premrov Bajuk B Babnik K Snoj T Milčinski L Pislak Ocepek M Škof M. Coumaphos residues in honey, bee brood, and beeswax after Varroa treatment. Apidologie. (2017) 48:588–98. 10.1007/s13592-017-0501-y

[55] Poirier L Jacquet P Plener L Masson P Daudé D Chabrière E. Organophosphorus poisoning in animals and enzymatic antidotes. Environ Sci Pollut Res. (2021) 28:25081–106. 10.1007/s11356-018-2465-5 29959732

[56] Volpato D Holzgrabe U. Designing hybrids targeting the cholinergic system by modulating the muscarinic and nicotinic receptors: a concept to treat Alzheimer's disease. Molecules. (2018) 23:3230. 10.3390/molecules23123230 30544533 PMC6320942

[57] Matsunami N Koizumi K Fukatsu C Yasuda K Fukatsu K. Efficacy of donepezil hydrochloride in canine cognitive dysfunction syndrome. J Anim Clin Med. (2010) 19:91–3. 10.11252/dobutsurinshoigaku.19.91

[58] Araujo JA Greig NH Ingram DK Sandin J de Rivera C Milgram NW. Cholinesterase inhibitors improve both memory and complex learning in aged beagle dogs. J Alzheimers Dis. (2011) 26:143–55. 10.3233/JAD-2011-110005 21593569 PMC4979003

[59] Becker RE Greig NH. Was phenserine a failure or were investigators mislead by methods? Curr Alzheimer Res. (2012) 9:1174–81. 10.2174/156720512804142912 23227991 PMC5182048

[60] Food Drug Administration. ARICEPT (Donepezil Hydrochloride) Tablets. (1996). Available online at: https://www.accessdata.fda.gov/drugsatfda_docs/label/2012/020690s035,021720s008,022568s005lbl.pdf (accessed October 26, 2022).

[61] Meden A Knez D Malikowska-Racia N Brazzolotto X Nachon F Svete J. Structure-activity relationship study of tryptophan-based butyrylcholinesterase inhibitors. Eur J Med Chem. (2020) 208:112766. 10.1016/j.ejmech.2020.112766 32919297

[62] Dewey CW Davies ES Xie H Wakshlag JJ. Canine cognitive dysfunction: pathophysiology, diagnosis, and treatment. Vet Clin North Am Small Anim Pract. (2019) 49:477–99. 10.1016/j.cvsm.2019.01.013 30846383

[63] Prpar Mihevc S Majdič G. Canine cognitive dysfunction and Alzheimer's disease - two facets of the same disease? Front Neurosci. (2019) 13:604. 10.3389/fnins.2019.00604 31249505 PMC6582309

[64] Stylianaki I Polizopoulou ZS Theodoridis A Koutouzidou G Baka R Papaioannou NG. Amyloid-beta plasma and cerebrospinal fluid biomarkers in aged dogs with cognitive dysfunction syndrome. J Vet Intern Med. (2020) 34:1532–40. 10.1111/jvim.15812 32557873 PMC7379053

[65] Schütt T Toft N Berendt M. Cognitive function, progression of age-related behavioral changes, biomarkers, and survival in dogs more than 8 years old. J Vet Intern Med. (2015) 29:1569–77. 10.1111/jvim.13633 26463980 PMC4895687

[66] Davis PR Head E. Prevention approaches in a preclinical canine model of Alzheimer's disease: benefits and challenges. Front Pharmacol. (2014) 5:47. 10.3389/fphar.2014.00047 24711794 PMC3968758

[67] Vitek MP Araujo JA Fossel M Greenberg BD Howell GR Rizzo SJS. Translational animal models for Alzheimer's disease: an Alzheimer's Association Business Consortium Think Tank. Alzheimers Dement (NY). (2021) 6:e12114. 10.1002/trc2.12114 33457489 PMC7798310

[68] Studzinski CM Araujo JA Milgram NW. The canine model of human cognitive aging and dementia: pharmacological validity of the model for assessment of human cognitive-enhancing drugs. Prog Neuropsychopharmacol Biol Psychiatry. (2005) 29:489–98. 10.1016/j.pnpbp.2004.12.014 15795058

[69] Košak U Brus B Knez D Šink R Žakelj S Trontelj J. Development of an *in-vivo* active reversible butyrylcholinesterase inhibitor. Sci Rep. (2016) 6:39495. 10.1038/srep39495 28000737 PMC5175178

[70] Zakošek Pipan M Prpar Mihevc S Štrbenc M Košak U Ilić IG Trontelj J. Treatment of canine cognitive dysfunction with novel butyrylcholinesterase inhibitor. Sci Rep. (2021) 11:18098. 10.1038/s41598-021-97404-2 34518582 PMC8438013

[71] Kim SS Lee SI Chung CJ Lee SC. The antagonistic effect of neostigmine on rocuronium-, clindamycin-, or both-induced neuromuscular blocking in the rat phrenic nerve-hemidiaphragm. Korean J Anest. (2011) 61:320–6. 10.4097/kjae.2011.61.4.320 22110886 PMC3219779

[72] Min CH Min YS Lee SJ Sohn UD. The comparative effects of aminoglycoside antibiotics and muscle relaxants on electrical field stimulation response in rat bladder smooth muscle. Arch Pharm Res. (2016) 39:863–70. 10.1007/s12272-016-0765-1 27260628

[73] Haake A Nguyen K Friedman L Chakkamparambil B Grossberg GT. An update on the utility and safety of cholinesterase inhibitors for the treatment of Alzheimer's disease. Expert Opin Drug Saf. (2020) 19:147–57. 10.1080/14740338.2020.1721456 31976781

[74] Marucci G Buccioni M Ben DD Lambertucci C Volpini R Amenta F. Efficacy of acetylcholinesterase inhibitors in Alzheimer's disease. Neuropharmacology. (2021) 190:108352. 10.1016/j.neuropharm.2020.108352 33035532

[75] Zadik-Weiss L Ritter S Hermush V Asher N Avital A Or R. Feline cognitive dysfunction as a model for Alzheimer's disease in the research of CBD as a potential treatment-a narrative review. J Cannabis Res. (2020) 2:43. 10.1186/s42238-020-00054-w 33526138 PMC7819322

[76] Robinson SR Bishop GM Lee HG Münch G. Lessons from AN 1792 Alzheimer vaccine: lest we forget. Neurobiol Aging. (2004) 25:609–15. 10.1016/j.neurobiolaging.2003.12.020 15172738

[77] Meden A Knez D Brazzolotto X Nachon F Dias J Svete J. From tryptophan-based amides to tertiary amines: optimization of a butyrylcholinesterase inhibitor series. Eur J Med Chem. (2022) 234:114248. 10.1016/j.ejmech.2022.114248 35299116

[78] Mazej T Knez D Meden A Gobec S Sova M. 4-Phenethyl-1-propargylpiperidine-derived dual inhibitors of butyrylcholinesterase and monoamine oxidase B. Molecules. (2021) 26:4118. 10.3390/molecules26144118 34299393 PMC8305717

[79] Sordo L Martini AC Houston EF Head E Gunn-Moore D. Neuropathology of aging in cats and its similarities to human Alzheimer's disease. Front Aging. (2021) 2:684607. 10.3389/fragi.2021.684607 35822024 PMC9261448

[80] Sordo L Gunn-Moore DA. Cognitive dysfunction in cats: update on neuropathological and behavioural changes plus clinical management. Vet Rec. (2021) 188:e3. 10.1002/vetr.3 34651755

